# Comparison between Two Radiological Methods for Assessment of Tooth Root Resorption: An In Vitro Study

**DOI:** 10.1155/2018/5152172

**Published:** 2018-03-04

**Authors:** Sabina Saccomanno, Pier Carmine Passarelli, Bruno Oliva, Cristina Grippaudo

**Affiliations:** Department of Dental Clinic, Catholic University of Sacred Heart of Rome, Rome, Italy

## Abstract

**Purpose:**

This study aims to verify the validity of the radiographic image and the most effective radiological techniques for the diagnosis of root resorption to prevent, cure, and reduce it and to verify if radiological images can be helpful in medical and legal situations.

**Methods:**

19 dental elements without root resorption extracted from several patients were examined: endooral and panoramic radiographs were performed, with traditional and digital methods. Then the root of each tooth was dipped into 3-4 mm of 10% nitric acid for 24 hours to simulate the resorption of the root and later submitted again to radiological examinations and measurements using the same criteria and methods.

**Results:**

For teeth with root resorption the real measurements and the values obtained with endooral techniques and digital sensors are almost the same, while image values obtained by panoramic radiographs are more distorted than the real ones.

**Conclusions:**

Panoramic radiographs are not useful for the diagnosis of root resorption. The endooral examination is, in medical and legal fields, the most valid and objective instrument to detect root resorption. Although the literature suggests that CBCT is a reliable tool in detecting root resorption defects, the increased radiation dosage and expense and the limited availability of CBCT in most clinical settings accentuate the outcome of this study.

## 1. Introduction

Histologically root resorption is an irreversible demineralization of the cementum (sometimes of the dentin) of the surface of the root of a tooth [1]. Diagnosis can be done by anamnestic data and careful clinical observation, but only radiological examinations are determinant, often exclusive, and usually conclusive. Causes of root resorption can be general or local: endocrine pathologies, significant oral dysfunctions, osteoporosis, traumas or external causes (orthodontic treatments), expanders, intrusion movements, aggressive or inappropriate orthodontic therapy, and therapy in patients with predisposition for root resorption (traumas, osteoporosis, or hypothyroidism) [2–4].

There are several theories about root resorption: Becks et al. [5, 6] wrote about hereditary transmission of the individual predisposition for root resorption. Rygh et al. [7] write about predisposition not only in different individuals, but also in the same person at different times, as hormone metabolical signals can change the osteoblastic/osteoclastic activity.

The age of the patient is important: the greatest orthodontic resorption is noticeable during adolescence when more orthodontic treatments are made and the apexes are already closed. Sameshima and Sinclair [8] stress that radicular resorption increases with age, especially in the anterior segment, corresponding to the incisors. Melsen et al. [9] assert that adults do not have the same cellular pool of young people and that is why forces applied to teeth through orthodontic treatment should be reduced, because the quantity of bone that must be resorbed in relation to a peculiar dental movement is also reduced.

Most authors [10] find no connection between sex and root resorption, although Brezniak and Wasserstein [11, 12] assert that females are more incline to idiopathic radicular reabsorption to the ratio of 3.7 : 1.

Specific radiological examinations can be valid documentation especially for orthodontists who, more than other specialists, are often subject to medical-legal jurisdiction concerning a contentious resorption of root. The radiologic examinations are actually an impartial permanent document that in the medical-legal field can be evaluated by several examiners, more independently than other subjective clinical evaluations [13, 14]. Radiologic examinations can reveal a pathology before the clinical symptoms appear, which is important, especially if an inappropriate orthodontic treatments can be avoided, if it could worsen the conditions of the teeth and their roots.

Teeth, because of their high density, are extremely radiopaque and radiographically well defined; therefore, endooral radiography (periapical radiographs) and extraoral radiography (orthopanoramic or OPG), based on both traditional and digital methods, can give useful information to the specialist [2, 15, 16]. Although the Cone Beam Computerized Tomography (CBCT) has become a very important tool in diagnostics, our study focused primarily on 2D digital analysis. Therefore we did not include CBCT analysis of root resorption in this study.

### 1.1. Traditional Method

#### 1.1.1. Orthopanoramic Technique (OPG)

It visualizes wide surfaces 13 × 18/18 × 24/24 × 30 cm and it is made by an orthopantomograph in which the X-ray origin and the film box rotate simultaneously while the patient remains still with his/her chin on the appropriate support [17]. It is useful as a first diagnostic orientation and offers a general image of teeth, arches, maxillary bones, paranasal sinuses and TMJ but, because of its approximate definition and dental overlap, it does not provide the same anatomical details obtained with endooral radiography.

#### 1.1.2. Endooral Technique

It visualizes smaller surfaces 2 × 3/3 × 4/4 × 5/5 × 7 cm and offers more accurate radiographic data and details about the dental alveoli. The exact image of the examined structure is not obtained, because the image is deformed by the angulations of rays that come from a single point but conic origin.

To reduce deformations, the film must be positioned parallel and near the structure to impress, so that the central ray of the beam can hit perpendicularly both the structure and the film.

### 1.2. Digital Method

This method is used for both the endooral (endooral RX) and extraoral (OPG) techniques.

Digital radiology assigns a numerical value to the different levels of X-ray absorption in the tissues and to differentiate the several tones of grey that compose the radiographic image. Digital radiology is divided into inline digital radiology, if the numerical image can be obtained directly from the patient, and into outline digital radiology, if the numerical image is obtained by a previously made diagram. The reduction in radiation dosage in digital radiology, the quality of images, and the postprocessing elaboration and optimization are all parts of an important progress in dental radiology.

This study aims to verify the validity of the radiographic image and the most effective radiological techniques for the diagnosis of rizalysis to prevent, cure, and reduce damage or reabsorption of the root. This study aims also to verify if radiological images can be helpful in medical and legal situations.

## 2. Materials and Methods

We examined a sample of 19 dental elements without root resorption extracted from several patients and for different reasons; each element has been measured in decimals of millimeter by caliper.

We used a long cone radiographic apparatus (70 kv and 7 mA) with a constant tension and constant times of exposure (0.12 sec for the traditional methods and 0.10 sec for digital sensors).

**Figure 1:**
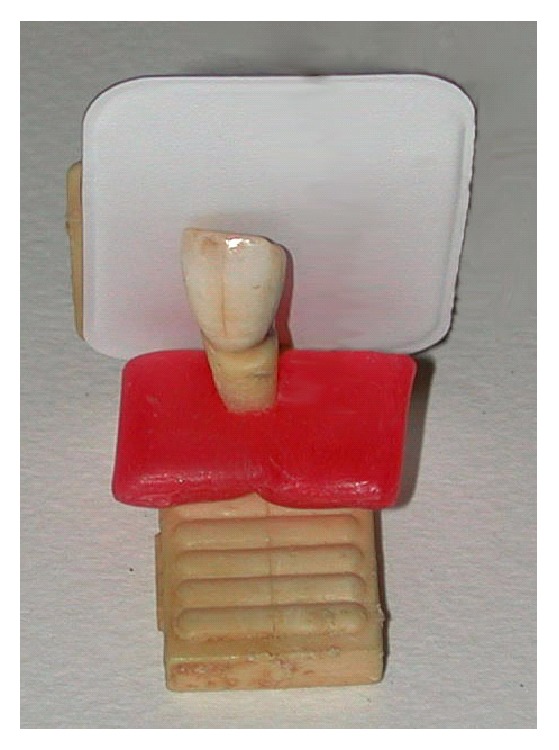
Extracted tooth on the endooral centering support.

We used a standardizing centering support (Figure 1) on which teeth have been positioned and fixed by orthodontic wax; then we placed the centering support 20 cm away from the X-ray origin and took radiographs of each tooth using both methods. The apex of the root and coronal border of each tooth were our reference marks and the measurements with a caliper were made after drawing the parallel tangent lines to these points.

**Figure 2:**
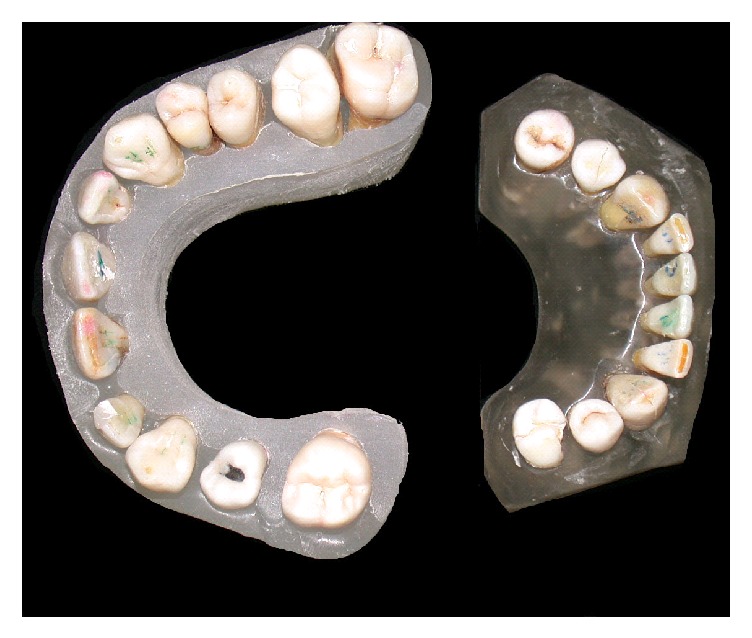
Simulated dental arch made of orthodontic resin, methacrylic orthocryl: occlusal vision.

We later created a simulated dental arch made of orthodontic resin, methacrylic orthocryl (Figure 2), and to recreate a thickness simulating soft tissues we used a common medical gauze. Then orthopanoramic radiographic examinations (OPG), a traditional method, were applied.

The examinations were made with a 60 Kv and 9.0 mA/15 sec orthopantomograph, because of the material used for the simulated dental arch. It was not possible to lower these parameters and so we decided to superimpose three films in the box to obtain three images and to have the possibility of choosing the best one. Digital measurements were made using a calibrated system from an image derived from a computerized program (Image J).

**Figure 3:**
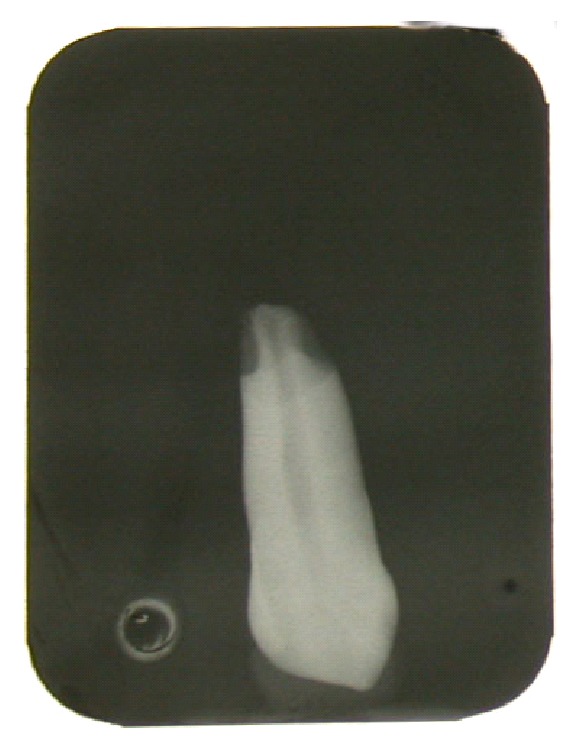
Traditional endooral X-ray: rizalysis.

**Figure 4:**
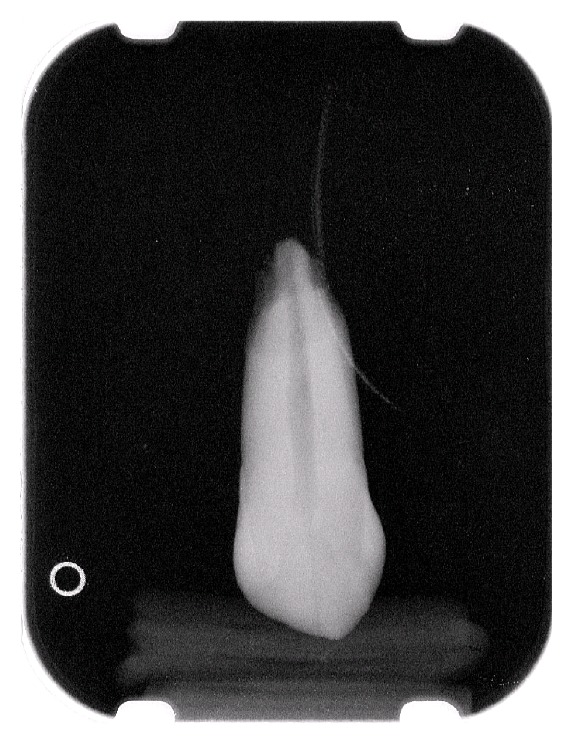
Digital endooral X-ray: rizalysis.

**Figure 5:**
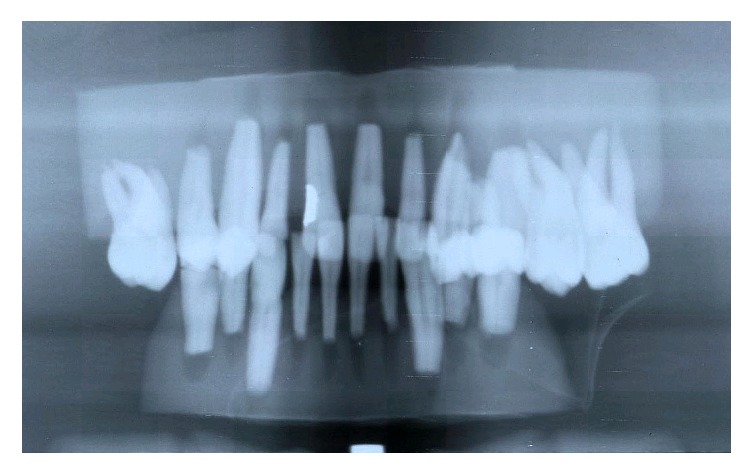
Panoramic radiograph: rizalysis.

Then the root of each tooth was dipped into 3-4 mm of 10% nitric acid for 24 hours to simulate of resorption of the root and later submitted again to radiological examinations and measurements using the same criteria and methods (Figures 3, 4, and 5). The measurements obtained were then statistically compared.

All the data collected was processed by a statistical program to calculate the percentage of the standard deviation of the values obtained with different radiological methods, before and after reabsorption of the root.

## 3. Results

Using the obtained data, recorded in Tables 1 and 2, we draw some curves whose observation led us to the following results:

- The most reliable radiological method is the endooral technique because the values we obtained are more homogeneous and more similar to the real ones.
- The extraoral technique (OPG) is the least reliable, as it gives a bigger image that distorts the original size. Plus, it does not offer precise anatomic details because of the presence of reinforcement protections that lowers the quantity of radiations needed to impress the image. The resulting tables show how the percentage of the deformation values has increased when compared to the endooral projections.
- If we compare both tables, we notice that the values of the teeth submitted to rizalysis and then analyzed with extraoral method (OPG) have increased image deformation.
- For teeth with root resorption the real values and the values obtained with endooral technique and digital sensors are almost the same, while image values obtained by extraoral methods are more distorted than the real ones.

## 4. Discussion

This study for the diagnosis of the radicular resorption has been made comparing various radiographic methods and techniques* in vitro* and shows the advantages and disadvantages of each method and technique.

The endooral technique provides a better qualitative and quantitative analysis than the orthopanoramic one. The limits are the absence of a standard process and the distortion of the endooral projections using the bisecting method: if the ray beam has a juxta-gingival or parallel orientation, the dental roots appear longer, while if the beam exceeds the apexes, the roots appear shorter.

The examinations by OPG provide less than neat images, with a bigger distorting enlargement and that is why OPG is not useful for the diagnosis of root resorption, but rather it is used to exclude other dental causes (agenesis, inclusion, or ectopy). It cannot be considered a valid diagnostic and medical-legal examination because at the level of the anterior part of the maxilla and the mandible, corresponding with the incisor teeth, there is a thinning of the tomographic section, exactly where the thickness should be as great as possible. If we combine all these shortcomings, the OPG exam becomes totally useless to assess root resorption. Therefore, studying root resorption using OPG, at the end of an orthodontic treatment, for instance, is not a very accurate method because of the distortion of the image and the variation of the dental inclination, which is also evident in the endooral projections [18, 19].

It is of paramount importance to have a wide thickness of the section examined, for the correct and full vision of the anterior elements on the OPG radiogram. This section must be as wide as possible to reproduce the total vertical dimension of maxillary and mandibular incisors because in the anterior region there is a thinning of the tomographic section that, in people with an increased interincisor angle, is more significant than the average, so there is a need for a particular image of these elements [20].

Moreover, if the OPG is executed with a digital method it is possible to have greater diagnostic precision for specific anatomic structures, whose interfaces are better shown by the border effect: superior and inferior frontal teeth, radicular apexes, nasal choanae floor of the maxillary sinuses, nasal and maxillary sinus septum, nasal septum, and mandibular condyles. The digital OPG examinations produce less distorted images and shades, so typical in the topographic method, with the dosage saving being also a positive aspect.

In this study we obtained very good images of the coronal and radicular structures by periapical X-rays and identified well the simulated radicular lesion. In addition, the compared values of the measurements are more similar to the real ones. For these reasons we think that the orthodontist should also prescribe, along with normal routine examinations, periapical endooral RX of incisors, canines, and premolars (these teeth are most subject to root resorption) with the X-ray tube positioner in place for the orthogonality of the rays in relation to the film and for the constant preservation of the right distance between teeth and X-ray origin [21].

Digital radiography gives us more definite images of crowns and roots using the wide grey scale and the possibility of elaborating the obtained data in postprocessing: the dose of radiation is low, the images are more reliable and immediately visualizable, and the operator can modify them to show details, enlarge them, measure, and easily file them to be used when necessary.

The conventional radiographic methods give bidimensional images and do not allow a proper vision of the lacunae of minor reabsorption. Since the Cone Beam Computerized Tomography (CBCT) might be useful for enhancing diagnosis of early root resorption, there is a need to compare the diagnostic accuracy of the CBCT images with digital periapical radiographs for assessing root defects. The CBCT is effective and reliable in detecting the presence of resorption lesions, although digital intraoral radiography results in an acceptable level of accuracy [22].

Although the literature suggests that CBCT is a reliable tool in detecting root resorption defects, it is rare that studies compare CBCT efficacy with intraoral digital 2D modalities. Kumar et al. [23] showed that examiners performed slightly weaker in the detection of root defects when using CBCT images compared with conventional periapical radiographs. The increased radiation dosage and expense and the limited availability of CBCT in most clinical settings accentuate the outcome of this study, which is to confirm that periapical radiographs are better at detecting root defects. However, when a full-mouth series or periapical radiographs are not available and a CBCT scan of the patient is already available, the CBCT data could be used to detect root resorption via 2D or 3D evaluation without additional radiation exposure. The lack of superiority of either imaging modality suggests that periapical or CBCT imaging can be used to identify root defects. However, because of the increased radiation exposure from CBCT, using CBCT for identifying defects should be considered with the caveat that CBCT data is already available for analysis [23]. Sousa Melo et al. [24] simulated “early stage” external root resorption lesions in the apical third of anterior teeth: they suggested there was a considerable difference between tomographic images with the most common voxel size used in orthodontics (0.4 mm) and those with a smaller voxel size (0.125 mm), suggesting that a more dedicated, high-resolution scan should be acquired when one intends to investigate the early stage of external root resorption during orthodontic treatment. This method could be the most reliable approach in detecting root resorption but the selection of voxel size increases the radiation exposure [25, 26]. Lima et al. [27] studied root resorption after dental trauma comparing periapical radiography and CBCT: they confirmed the superiority of CBCT in identifying it. However, they also reported that CBCT should not be used routinely for diagnosing root resorption, but it is recommended when lesion is suspected and more information about the shape of the defects is needed. All exposure to ionizing radiation should follow the “as low as reasonably achievable” principle and for this reason, the selection criteria and the parameters for each CBCT scan protocol should be strict and follow the respective clinical indication. Some limitations are associated with this study: diagnostic test may be affected by observer performance and experience, hardware and software specification, and viewing conditions; in in vitro study there are no nuances of human soft tissue attenuation artifacts. One limitation of our study was the small sample size. The size was based on previous root resorption research [22, 23]. Further studies with a bigger sample size than ours are requested to confirm these results.

## 5. Conclusions

Radiologic examinations can notice the pathology before the clinical symptoms appear, especially useful if we can avoid inappropriate orthodontic treatments which could worsen the conditions of the teeth and their roots.

All teeth submitted for orthodontic treatment may have a very small amount of resorption that is clinically insignificant and radiologically invisible. This kind of resorption usually does not influence the functional activity and life of the tooth because it stops as soon as the active treatment is finished. This range of resorption of root can be considered a small price to pay if compared with all the advantages of a well-done orthodontic treatment.

From our study, it may be inferred that the endooral examination is, in medical and legal fields, the most valid and objective instrument to detect root resorption. Although a more advanced method such as the CBCT may yield similar results, the device is not suitable for most working realities or even for most clinics worldwide, confirming the traditional endooral X-ray as the best option to examine root resorption in a wide variety of situations, especially in case of elective orthodontic treatment.

## Figures and Tables

**Table 1:** Tooth length without tooth resorption.

Tooth number	Real tooth length	Radiological measurements using traditional endooral X-rays		Radiological measurements using panoramic radiographs		Radiological measurements using digital endooral X-rays	
	Total	Var.%	Total	Var.%	Total	Var.%	Total
11	24.84	4.31%	25.91	8.86%	27.04	3.38%	25.68
12	21.80	5.73%	23.05	11.56%	24.32	5.47%	22.99
13	28.06	4.49%	29.32	10.83%	31.10	5.63%	29.64
14	24.60	1.38%	24.94	9.02%	26.82	0.41%	24.70
21	24.86	4.42%	25.96	13.19%	28.14	2.96%	25.60
22	22.50	3.60%	23.31	12.53%	25.32	12.94%	25.41
23	28.24	4.92%	29.63	11.19%	31.40	4.25%	29.44
24	20.20	7.13%	21.64	5.45%	21.30	5.64%	21.34
25	22.60	3.54%	23.40	11.50%	25.20	5.78%	23.91
31	21.80	3.67%	22.60	13.30%	24.70	3.04%	22.46
32	22.16	4.74%	23.21	18.41%	26.24	1.89%	22.58
33	26.82	2.68%	27.54	23.79%	33.20	0.74%	27.02
34	19.74	6.69%	21.06	21.58%	24.00	1.89%	20.11
35	20.14	2.78%	20.70	11.22%	22.40	1.54%	20.45
41	21.30	9.86%	23.40	16.81%	24.88	5.32%	22.43
42	22.38	3.22%	23.10	13.14%	25.32	0.18%	22.42
43	27.70	4.12%	28.84	15.81%	32.08	0.36%	27.80
44	20.76	4.91%	21.78	13.68%	23.60	0.29%	20.82
45	20.20	4.46%	21.10	20.89%	24.42	5.63%	21.34

**Table 2:** Tooth length with rizalysis.

Tooth number	Real tooth length	Radiological measurements using traditional endooral X-rays		Radiological measurements using panoramic radiographs		Radiological measurements using digital endooral X-rays	
	Total	Var.%	Total	Var.%	Total	Var.%	Total
11	19.72	2.03%	20.12	12.07%	22.10	1.70%	20.06
12	18.72	3.74%	19.42	18.59%	22.20	0.56%	18.82
13	23.88	0.42%	23.98	13.15%	27.02	0.06%	23.90
14	15.25	13.18%	17.26	32.59%	20.22	1.39%	15.46
21	19.62	2.55%	20.12	18.25%	23.20	2.91%	20.19
22	17.38	1.73%	17.68	12.08%	19.48	1.06%	17.56
23	22.24	2.11%	22.71	8.27%	24.08	32.38%	29.44
24	15.25	4.26%	15.90	23.80%	18.88	1.39%	15.46
25	18.22	1.43%	18.48	21.41%	22.12	1.02%	18.41
31	16.72	0.60%	16.82	22.61%	20.50	0.42%	16.79
32	17.62	0.57%	17.72	16.40%	20.51	0.46%	17.70
33	21.40	0.93%	21.60	20.19%	25.72	0.47%	21.50
34	13.36	4.79%	14.00	16.62%	15.58	0.49%	13.43
35	15.21	1.78%	15.48	17.69%	17.90	0.36%	15.27
41	17.54	2.05%	17.90	25.43%	22.00	0.57%	17.64
42	15.70	2.55%	16.10	16.94%	18.36	0.36%	15.76
43	23.29	0.64%	23.44	14.21%	26.60	0.36%	23.37
44	15.50	2.84%	15.94	18.45%	18.36	0.40%	15.56
45	16.20	6.79%	17.30	21.11%	19.62	1.58%	16.46
